# Supplementary material for: Repurposing bunamidine hydrochloride as a potent antimicrobial agent targeting vancomycin-resistant Enterococcus membranes
Source: mSystems. 2025 Oct 29;10(11):e00425-25. doi: 10.1128/msystems.00425-25 (PMC12625711; doi:10.1128/msystems.00425-25)
Supplement: Supplemental material — Supplemental figures and tables. [file msystems.00425-25-s0001.docx]

**Supplementary Information**

Supplementary Table 1 |Bacterial strains used in this study

| Strain | Species | Resistance Type | Source |
| --- | --- | --- | --- |
| ATCC 29212 | *E. faecalis* | Type strain,VSE | American Type Culture Collection (ATCC), USA |
| ATCC 51299 | *E. faecalis* | Type strain,VRE | American Type Culture Collection (ATCC), USA |
| EFA2433084 | *E. faecalis* | Clinical isolate,VSE | Affiliated Changsha Hospital, Xiangya School of Medicine, Hunan, China |
| EFA2433159 | *E. faecalis* | Clinical isolate,VSE | Affiliated Changsha Hospital, Xiangya School of Medicine, Hunan, China |
| EFA2433254 | *E. faecalis* | Clinical isolate,VSE | Affiliated Changsha Hospital, Xiangya School of Medicine, Hunan, China |
| EFA24330726 | *E. faecalis* | Clinical isolate,VSE | Affiliated Changsha Hospital, Xiangya School of Medicine, Hunan, China |
| ATCC 19434 | *E. faecium* | Type strain,VSE | American Type Culture Collection (ATCC), USA |
| U101 | *E. faecium* | Clinical isolate,VRE | ^26^ |
| SRYEFM1 | *E. faecium* | Clinical isolate,VRE | Donated by Xie Liangyi (Hunan Provincial People's Hospital, China) |
| SRYEFM13 | *E. faecium* | Clinical isolate,VRE | Donated by Xie Liangyi (Hunan Provincial People's Hospital, China) |
| SRYEFM2 | *E. faecium* | Clinical isolate,VRE | Donated by Xie Liangyi (Hunan Provincial People's Hospital, China) |
| SRYEFM5 | *E. faecium* | Clinical isolate,VRE | Donated by Xie Liangyi (Hunan Provincial People's Hospital, China) |
| SRYEFM9 | *E. faecium* | Clinical isolate,VRE | Donated by Xie Liangyi (Hunan Provincial People's Hospital, China) |
| SRYEFM6 | *E. faecium* | Clinical isolate,VRE | Donated by Xie Liangyi (Hunan Provincial People's Hospital, China) |
| SRYEFM15 | *E. faecium* | Clinical isolate,VRE | Donated by Xie Liangyi (Hunan Provincial People's Hospital, China) |

Supplementary Table 2 | *In vitro* antibacterial activity of BUN against representative Gram-positive and Gram-negative pathogens

| Strains | MIC(μg/mL) | MBC(μg/mL) | Source |
| --- | --- | --- | --- |
| \| Methicillin-resistant *Staphylococcus aureus* (MRSA) \| \| --- \| | | | |
| ATCC 43300 | 4 | 8 | American Type Culture Collection (ATCC), USA |
| USA300 | 4 | 8 | American Type Culture Collection (ATCC), USA |
| SAJ1 | 4 | 8 | the Third Xiangya Hospital of Central South University, Hunan, China |
| SA1901 | 4 | 8 | the Third Xiangya Hospital of Central South University, Hunan, China |
| Methicillin-sensitive *Staphylococcus aureus* (MSSA) | | | |
| LZB1 | 8 | 8 | Donated by Zhen Luo (the Third Xiangya Hospital of Central South University,Hunan, China) |
| \| *Staphylococcus epidermidis* (SE⁺) \| \| --- \| | | | |
| RP62A | 4 | 4 | Donated by Di Qu (Shanghai Medical College, Fudan University, China) |
| \| *Escherichia coli* \| \| --- \| | | | |
| ATCC 25922 | 32 | 32 | Donated by Juncai Luo (Tiandiren Biotech, China) |
| \| *Acinetobacter baumannii* \| \| --- \| | | | |
| ATCC 19606 | 32 | 32 | American Type Culture Collection (ATCC), USA |
| \| *Pseudomonas aeruginosa* \| \| --- \| | | | |
| PAO1 | >32 | >32 | Donated by Minqiang Qiao (College of Life Sciences, Nankai University, China) |
| \| *Klebsiella pneumoniae* \| \| --- \| | | | |
| ATCC 700603 | >32 | >32 | Donated by Juncai Luo (Tiandiren Biotech, China) |

SE⁺, biofilm-forming phenotype. MIC, minimum inhibitory concentration. MBC,minimum bactericidal concentration

Supplementary Table 3 |Pharmacokinetic parameters of BUN

| Route | mg/kg | T_1/2_(h) | T_max_(h) | C_max_(μ g/mL) | AUC(0~t) | AUC(0~∞） | MRT(0~t) | MRT(0~∞) | C0 | Vss | Vz | Cl | F(%) |
| --- | --- | --- | --- | --- | --- | --- | --- | --- | --- | --- | --- | --- | --- |
|  |  |  |  |  | h*μg/mL | h*μg/mL | h | h | μg/mL | L/kg | L/kg | mL/min/kg |  |
| i.v. | 10 | 5.06±1.63 | 0.11±0.07 | 1.25±0.58 | 3.61±0.96 | 3.76±1.05 | 4.53±0.85 | 5.63±1.75 | 0.05±0.00 | 1.54±0.60 | 20.55±8.01 | 47.73±14.81 | ‐ |
| s.c. | 30 | 5.96±3.13 | 0.51±0.40 | 0.74±0.60 | 1.92±1.53 | 3.13±2.37 | 3.43±2.14 | 9.15±4.63 | ‐ | ‐ | ‐ | ‐ | 27.80±22.45 |
| i.p. | 30 | 7.85±4.48 | 0.15±0.17 | 1.60±0.87 | 5.70±3.08 | 6.64±3.25 | 5.98±2.73 | 10.79±6.62 | ‐ | ‐ | ‐ | ‐ | 58.95±33.22 |

### ****
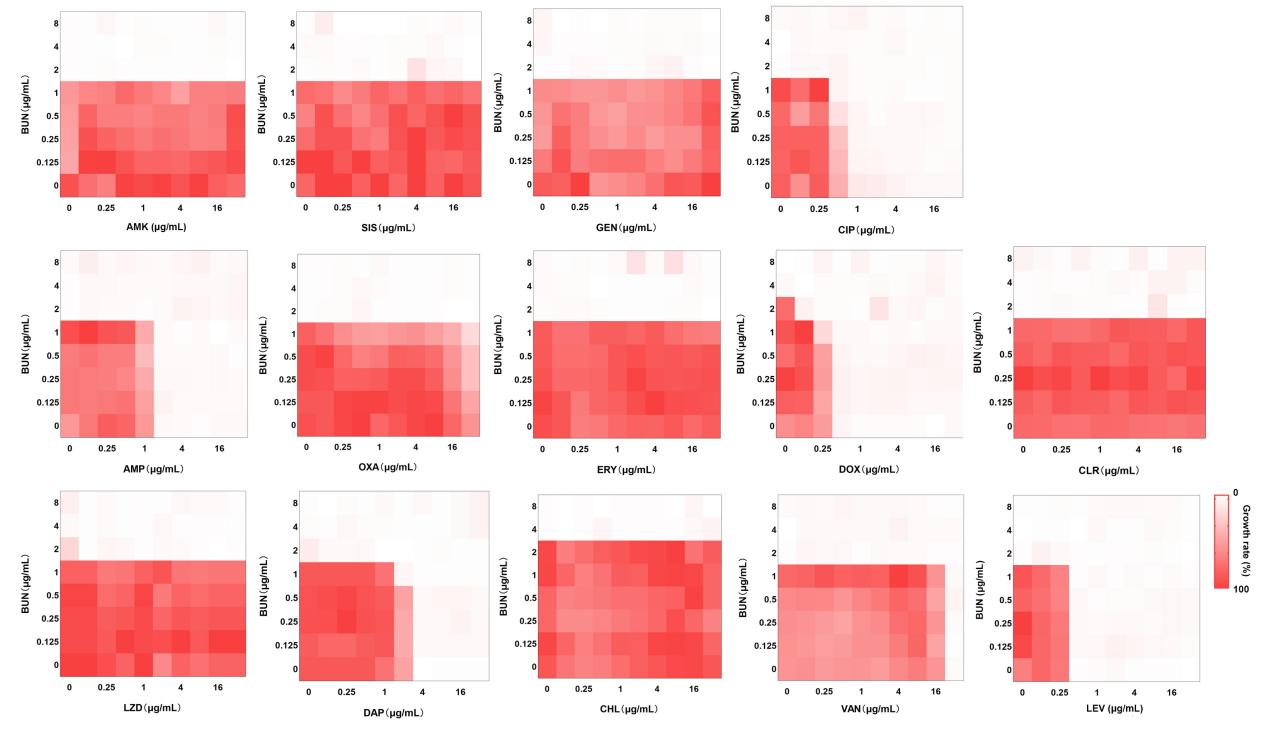
****

### Figure S1 | The combinational effects between BUN and conventional antibiotics assessed by checkerboard assay.


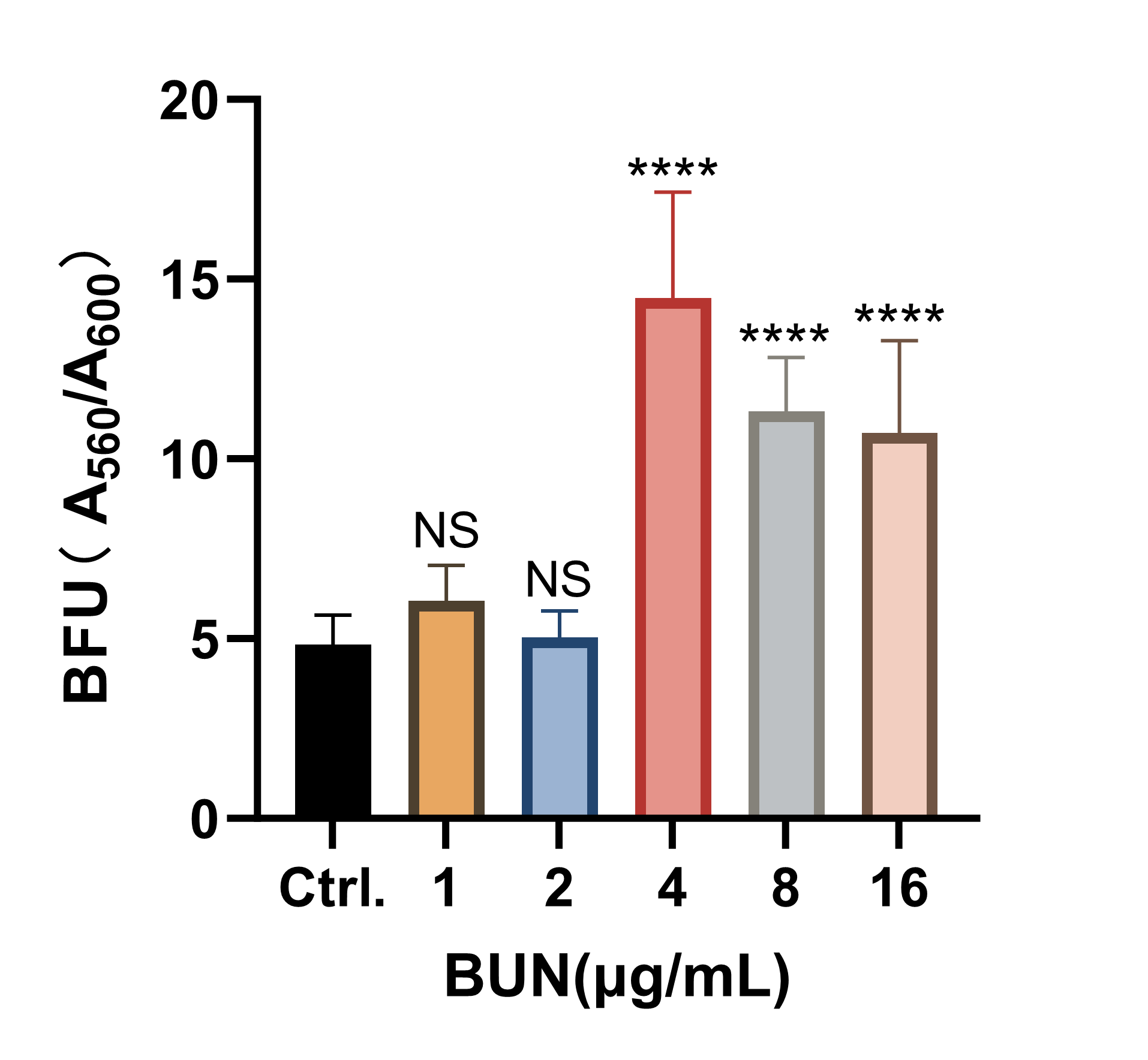


Figure S2 |Normalized biofilm biomass (biofilm formation units, BFU) of *E. faecalis* ATCC 51299 following treatment with BUN at indicated concentrations. BFU values were calculated as the ratio of biofilm biomass (A_560nm_) to planktonic growth (A_600nm_) to account for growth-dependent effects. Data represent mean ± SD from three independent experiments. Statistical significance was determined by one-way ANOVA with Dunnett’s post hoc test compared to the control group. NS, not significant; *****P*< 0.0001.

###
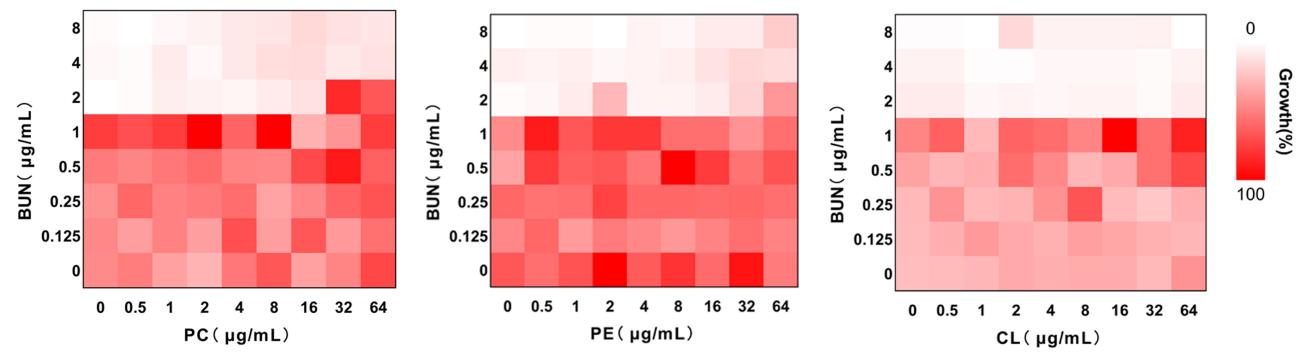


### Figure S3 |Competitive inhibition assays of BUN with exogenous membrane phospholipids of PE, PC, and CL against *E. faecalis* ATCC 51299.


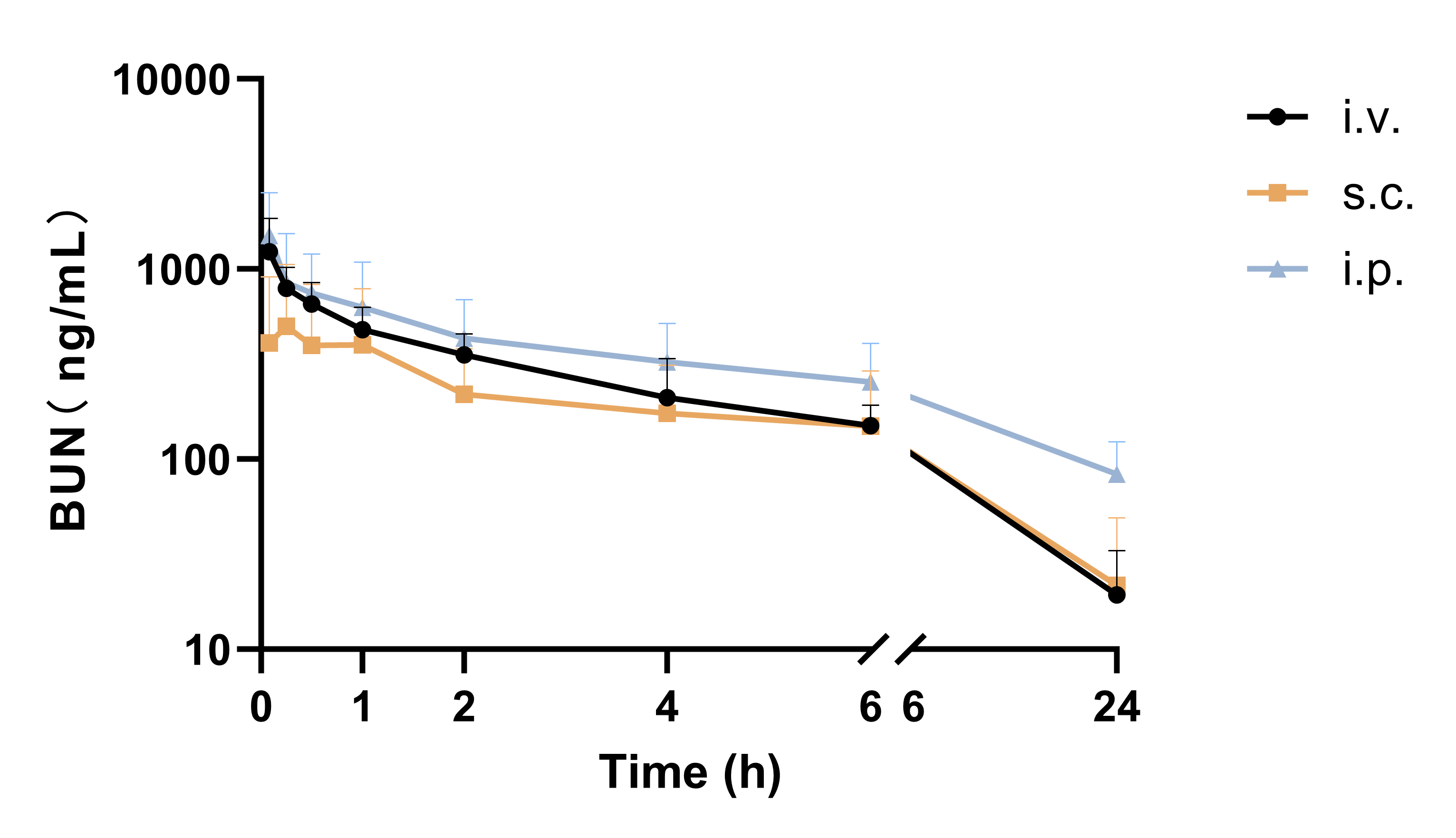


Figure S4 |Pharmacokinetic profile of BUN following different administration routes. Plasma concentration of BUN (ng/mL) over time (h) after intravenous (i.v.), subcutaneous (s.c.), and intraperitoneal (i.p.) injections. Data are expressed as mean ± standard deviation (n = 6). The pharmacokinetic curve shows the concentration-time profile of BUN for each administration route, highlighting the differences in drug distribution and elimination rates.

###
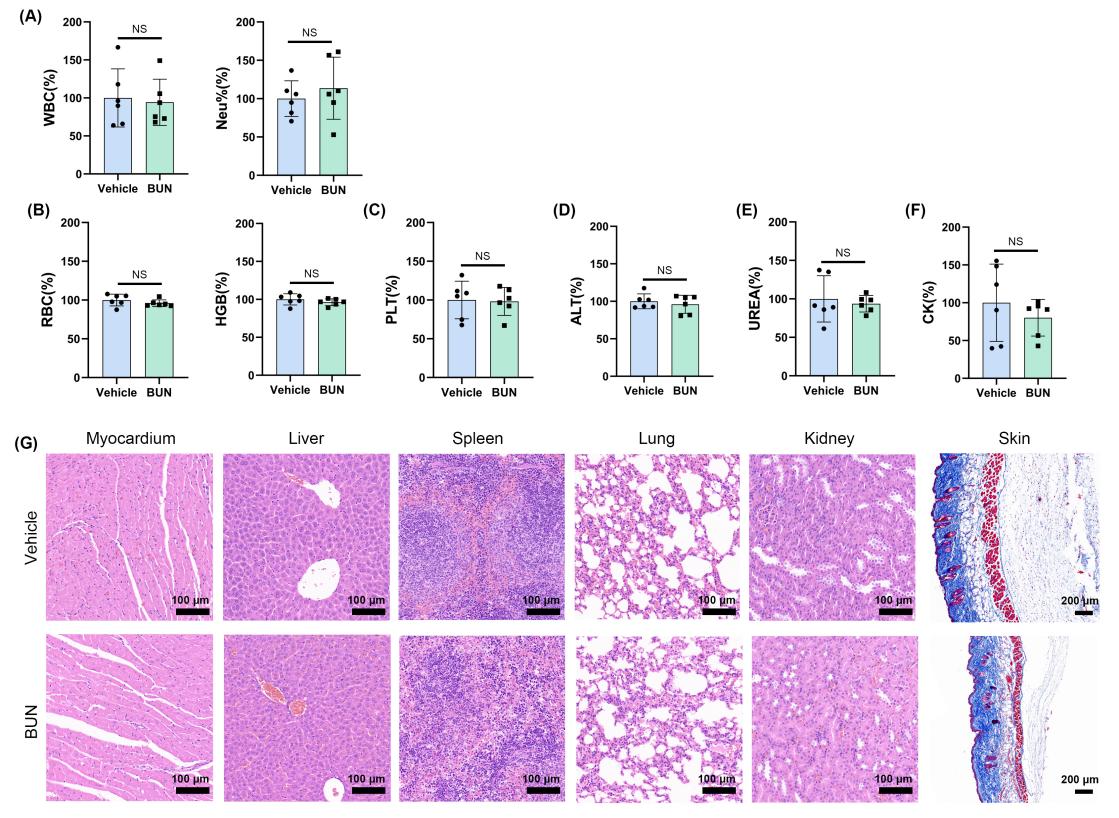


### Figure S5 | Acute *in vivo* toxicity by BUN in a mouse model. (A) Inflammatory response indicators quantification of the white blood cell (WBC) count and neutrophil percentage quantification (Neu%). (B) Erythrocyte-related hematological parameters, including red blood cell (RBC) count and hemoglobin (HGB) quantification. (C) Platelet (PLT) count, assessing potential coagulation abnormalities following BUN treatment. (D-F) Serum biochemical markers evaluating organic function. Alanine aminotransferase (ALT) for liver function (D), blood urea nitrogen (UREA) for kidney function (E), and creatine kinase (CK) for cardiac function (F). (G) Histopathological analysis of major organs. H&E staining was performed on myocardium, liver, spleen, lung, and kidney sections to assess potential tissue damage, while Masson staining was used to evaluate collagen deposition in skin tissues.
